# Supplementary material for: Mapping the evolution of fertility support policies in China: A content and instrumental analysis
Source: PLoS One. 2025 Oct 9;20(10):e0332137. doi: 10.1371/journal.pone.0332137 (PMC12510515; doi:10.1371/journal.pone.0332137)
Supplement: S1 Appendix — (ZIP) [file pone.0332137.s001.zip › S1 Appendix. 226 original policy documents/190-国家发展改革委、住房城乡建设部关于加强城镇老旧小区改造配套设施建设的通知(FBM-CLI-4-5055864).doc]

国家发展改革委、住房城乡建设部关于加强城镇老旧小区改造配套设施建设的通知

**发布部门：** [国家发展和改革委员会(含原国家发展计划委员会、原国家计划委员会)](https://www.pkulaw.com/law/chl?Aggs.IssueDepartment=60301) [住房和城乡建设部](https://www.pkulaw.com/law/chl?Aggs.IssueDepartment=60238) [机构沿革](javascript:void(0))

**发文字号：**发改投资〔2021〕1275号

**发布日期：**2021.09.02

**实施日期：**2021.09.02

**时效性：** [现行有效](https://www.pkulaw.com/law/chl?Aggs.TimelinessDic=01)

**效力级别：** [部门规范性文件](https://www.pkulaw.com/law/chl?Aggs.EffectivenessDic=XE0302)

**法规类别：** [建设综合规定](https://www.pkulaw.com/law/chl?Aggs.Category=04901)

国家发展改革委、住房城乡建设部关于加强城镇老旧小区改造配套设施建设的通知
（发改投资〔2021〕1275号）

各省、自治区、直辖市及计划单列市、新疆生产建设兵团发展改革委、住房城乡建设厅（住房城乡建设委、建设和交通委、建设局）：
　　加强城镇老旧小区改造配套设施建设，关乎人民群众生命财产安全，关乎满足人民群众美好生活需要，是“我为群众办实事”的一项生动实践。为贯彻落实党中央、国务院决策部署，加强城镇老旧小区改造配套设施建设与排查处理安全隐患相结合工作，现将有关要求通知如下：
　　一、加强项目储备
　　**（一）**进一步摸排城镇老旧小区改造配套设施短板和安全隐患。结合住房和城乡建设领域安全隐患排查整治工作，认真摸排2000年底前建成的需改造城镇老旧小区存在的配套设施短板，组织相关专业经营单位，联合排查燃气、电力、排水、供热等配套基础设施以及公共空间等可能存在的安全隐患；重点针对养老、托育、停车、便民、充电桩等设施，摸排民生设施缺口情况。

　　**（二）**科学编制年度改造计划。将安全隐患多、配套设施严重缺失、群众改造意愿强烈的城镇老旧小区，优先纳入年度改造计划，做到符合改造对象范围的老旧小区应入尽入。编制老旧小区改造方案时，把存在安全隐患的燃气、电力、排水、供热等设施，养老、托育、停车、便民、充电桩等民生设施，作为重点内容优先改造。

　　**（三）**规范履行审批程序。依法合规办理审批、核准、备案以及建设许可等手续。市县人民政府组织有关部门联合审查城镇老旧小区改造方案的，各相关部门应加强统筹、责任共担，避免顾此失彼；涉及燃气、电力、排水、供热等安全隐患改造内容，应确保安全审查不漏项。
　　二、强化资金保障

　　**（四）**政府投资重点保障。中央预算内投资全部用于城镇老旧小区改造配套设施建设项目。各地应统筹地方财力，重点安排消除城镇老旧小区各类安全隐患、提高排水防涝能力、完善养老托育设施、建设停车场和便民设施等城镇老旧小区配套设施改造内容。城镇老旧小区改造资金，积极支持消除安全隐患。

　　**（五）**落实专业经营单位责任。督促引导供水、排水、燃气、电力、供热等专业经营单位履行社会责任，将需改造的水电气热信等配套设施优先纳入年度更新改造计划，并主动与城镇老旧小区年度改造计划做好衔接；落实出资责任，优先安排老旧小区配套设施改造资金；落实安全责任，加强施工和运营维护力量保障，消除安全隐患。

　　**（六）**推动多渠道筹措资金。推动发挥开发性、政策性金融支持城镇老旧小区改造的重要作用，积极争取利用长期低成本资金，支持小区整体改造项目和水电气热等专项改造项目。鼓励金融机构参与投资地方政府设立的老旧小区改造等城市更新基金。对养老托育、停车、便民市场、充电桩等有一定盈利的改造内容，鼓励社会资本专业承包单项或多项。按照谁受益、谁出资原则，积极引导居民出资参与改造，可通过直接出资、使用（补建、续筹）住宅专项维修资金、让渡小区公共收益等方式落实。
　　三、加强事中事后监管

　　**（七）**加强项目实施工程质量安全监管。切实加强城镇老旧小区改造项目监管，项目行业主管部门严格落实日常监管责任，监管责任人应做到开工到现场、建设到现场、竣工到现场，发现问题督促及时解决。建设单位严格落实首要责任，严格按批复的建设内容和工期组织建设，保障工程项目质量安全；勘察设计单位应认真踏勘小区及周边设施情况，排查安全隐患，在改造方案中统筹治理；施工单位应严格按标准规范施工，确保施工质量和安全；监理单位应认真履行监理职责，特别是加强对相关设施安全改造的监督检查。

　　**（八）**强化项目建设统筹协调。将城镇老旧小区改造与城市更新以及排水、污水处理、燃气、电力等市政管网设施建设，养老、托育、停车等公共服务设施建设，体育彩票、福利彩票等各类专项资金支持建设的体育健身、无障碍等设施建设有机结合，统筹安排城镇老旧小区改造、防洪排涝、治污、雨水资源化利用、市政建设等工程，优化空间布局和建设时序，避免反复开挖。

　　**（九）**严格组织项目竣工验收。项目建成后，各级发展改革、住房和城乡建设部门应督促各有关方面，按照国家有关规定组织竣工验收，将安全质量作为竣工验收的重要内容。鼓励相关各方进行联合验收。安全质量达到规定要求的，方可通过竣工验收；安全质量未达到要求、仍存在隐患的要及时整改达标，否则不得通过竣工验收。
　　四、完善长效管理机制

　　**（十）**压实地方责任。各级城市（县）应切实履行安全管理主体责任，抓紧建立完善燃气、电力、排水、供热等市政设施管理制度。落实相关部门责任，按照职责开展安全监督检查。压实专业经营单位责任，按照有关规定开展安全巡查和设施管养。

　　**（十一）**充分发挥党建引领作用。推动建立党组织领导下的社区居委会、业主委员会、物业服务公司等广泛参与、共商事务、协调互动的社区管理新机制，推进社区基层治理体系和治理能力现代化，共同维护改造成果。

　　**（十二）**推行物业专业化管理。城镇老旧小区完成改造后，有条件的小区通过市场化方式选择专业化物业服务公司接管；引导将相关配套设施产权依照法定程序移交给专业经营单位，由其负责后续维护管理。建立健全住宅专项维修资金归集、使用及补建续筹制度。拓宽资金来源渠道，统筹公共设施经营收益等业主共有收入，保障城镇老旧小区后续管养资金需求。
　　五、其他事项
　　自2021年起，保障性安居工程中央预算内投资专项严格按照有关专项管理办法规定，支持小区内和小区周边直接相关的配套设施建设，不支持单独的城镇污水处理设施及配套管网建设。各地方要严格按要求将中央预算内投资分解落实到具体项目。2021年已分解落实的具体项目中，不符合要求的应及时调整并报国家发展改革委备案。
　　各级发展改革、住房和城乡建设部门要高度重视城镇老旧小区改造，加强城镇老旧小区改造配套设施建设与排查处理安全隐患相结合工作，强化项目全过程管理，强化事中事后监管，节约集约规范用好中央预算内投资，加快推进城镇老旧小区改造配套设施建设，切实提高人民群众安全感、获得感、幸福感。
　　特此通知。

国家发展改革委
　　住房城乡建设部
　　2021年9月2日

© 北大法宝：（[www.pkulaw.com](https://www.pkulaw.com/)) 专业提供法律信息、法学知识和法律软件领域各类解决方案。北大法宝为您提供丰富的参考资料，正式引用法规条文时请与标准文本核对。
欢迎查看所有[产品和服务](https://www.pkulaw.com/helps/2.html)。法宝快讯：[如何快速找到您需要的检索结果？](https://www.pkulaw.com/helps/70.html)[法宝](https://www.pkulaw.com/helps/69.html)V6有何新特色？

扫描二维码阅读原文

**原文链接：**<https://www.pkulaw.com/chl/b52d53e587032e4fbdfb.html>
